# LED-based optoacoustic tomography of mice

**DOI:** 10.1117/1.JBO.30.4.040501

**Published:** 2025-04-11

**Authors:** Sandeep Kumar Kalva, Xiang Liu, Xosé Luís Deán-Ben, Lin Tang, Daniel Razansky

**Affiliations:** a University of Zurich, Institute of Pharmacology and Toxicology, Institute for Biomedical Engineering, Faculty of Medicine, Zurich, Switzerland; b ETH Zurich, Institute for Biomedical Engineering, Department of Information Technology and Electrical Engineering, Zurich, Switzerland; c Indian Institute of Technology Bombay, Department of Biosciences and Bioengineering, Mumbai, Maharashtra, India

**Keywords:** light-emitting diode, whole-body imaging, optoacoustic/photoacoustic tomography, affordable light sources

## Abstract

**Significance:**

Optoacoustic tomography systems commonly employ bulky and expensive solid-state laser sources readily capable of generating dozens of millijoules of optical energy per pulse. Light-emitting diodes (LEDs) may offer a significantly more affordable and compact solution with excellent pulse-to-pulse stability. Yet, the optical design must optimize the energy density delivered to the target, given the relatively low per-pulse energy output of LEDs.

**Aim:**

We exploit a full-view LED-based optoacoustic tomography (FLOAT) configuration for *in vivo* imaging of mice.

**Approach:**

The system features panoramic light illumination delivering 0.48 mJ of total per-pulse energy with an array of 160 LEDs stacked into a cylindrically focused circular ultrasound array transducer.

**Results:**

We characterize the imaging performance of the FLOAT system in tissue-mimicking phantoms, subsequently demonstrating its ability for *in vivo* cross-sectional mouse imaging.

**Conclusions:**

It is anticipated that the compact, low-cost FLOAT imaging system will open up new venues in resource-limited settings for studying large-scale biodynamics such as pharmacokinetics and biodistribution of molecular agents and drugs on a whole-body level.

## 1 Introduction

Preclinical imaging plays an important role in studying disease pathophysiology and monitoring the efficacy of potential treatments.^1^^,^^2^ Owing to the development of transgenic and gene knockout/in techniques, small rodents are extensively used as animal models.^2^ Downscaling of several clinical imaging modalities, such as computed tomography (CT),^3^^,^^4^ magnetic resonance imaging (MRI),^5^^,^^6^ positron emission tomography (PET),^7^^,^^8^ and pulse-echo ultrasound (US),^9^^,^^10^ have greatly impacted preclinical studies. Other approaches based on optical contrast have further been developed for whole-body functional and molecular imaging.^11^^–^^14^ Albeit optical imaging techniques are free of ionizing radiation and have the particular advantage of rich functional and molecular contrast, they suffer from poor resolution when applied beyond the superficial areas in optically opaque mammalian tissues. Recently, optoacoustic tomography (OAT) has been gaining popularity in preclinical and clinical research^15^^–^^17^ due to its unique ability to combine rich optical contrast with high spatial resolution enabled by US beamforming in deep tissues. In addition, OAT systems offer fast imaging capabilities at frame rates of hundreds to thousands of hertz, which is important when observing rapid biodynamics.^18^

Conventional OAT systems employ Q-switched Nd:YAG pumped tunable optical parametric oscillator (OPO) or dye lasers that deliver high-energy laser pulses (typically in the range of 10 to 200 mJ) for efficient generation of US waves within biological tissue.^19^ These Nd:YAG-based lasers are bulky, heavy, and expensive. A vibration–isolation optical table is often needed to house those lasers to deliver stable energy output, making them difficult to move between locations.^20^ Portable OPO lasers have become available in recent years,^21^^,^^22^ but their cost remains prohibitive for widespread use. Recently, alternative light sources such as pulsed laser diodes (PLDs)^23^^–^^25^ and light-emitting diodes (LEDs)^26^^–^^28^ have gained attention owing to their compact and portable footprint, affordability, and high pulse repetition rates reaching several kHz.^29^ The non-coherent nature of LEDs offers a much safer and cost-effective alternative for OAT implementations, particularly in resource-limited and clinical settings. Generally, the pulse energies delivered by individual LED light sources are very low in the range of a few μJ, which may result in unacceptable signal-to-noise ratio (SNR) levels. Nevertheless, stacked arrays of LEDs deliver higher pulse energies up to a few 100s of μJ.^30^^,^^31^

Here, we exploit a full-view LED-based optoacoustic tomography (FLOAT) configuration for whole-body imaging of mice. FLOAT has the advantage of panoramic light illumination with full-view optoacoustic signal detection in 360 deg. It is based on custom-made stacked LED arrays delivering a total per-pulse energy of 0.48 mJ around the imaging object placed at the center of the circular full-ring ultrasound array transducer. This transducer plays a crucial role in circumventing limited-view artifacts and enhances the effective field-of-view (FOV) and image quality for cross-sectional imaging.

## 2 Methods

**Fig. 1:**
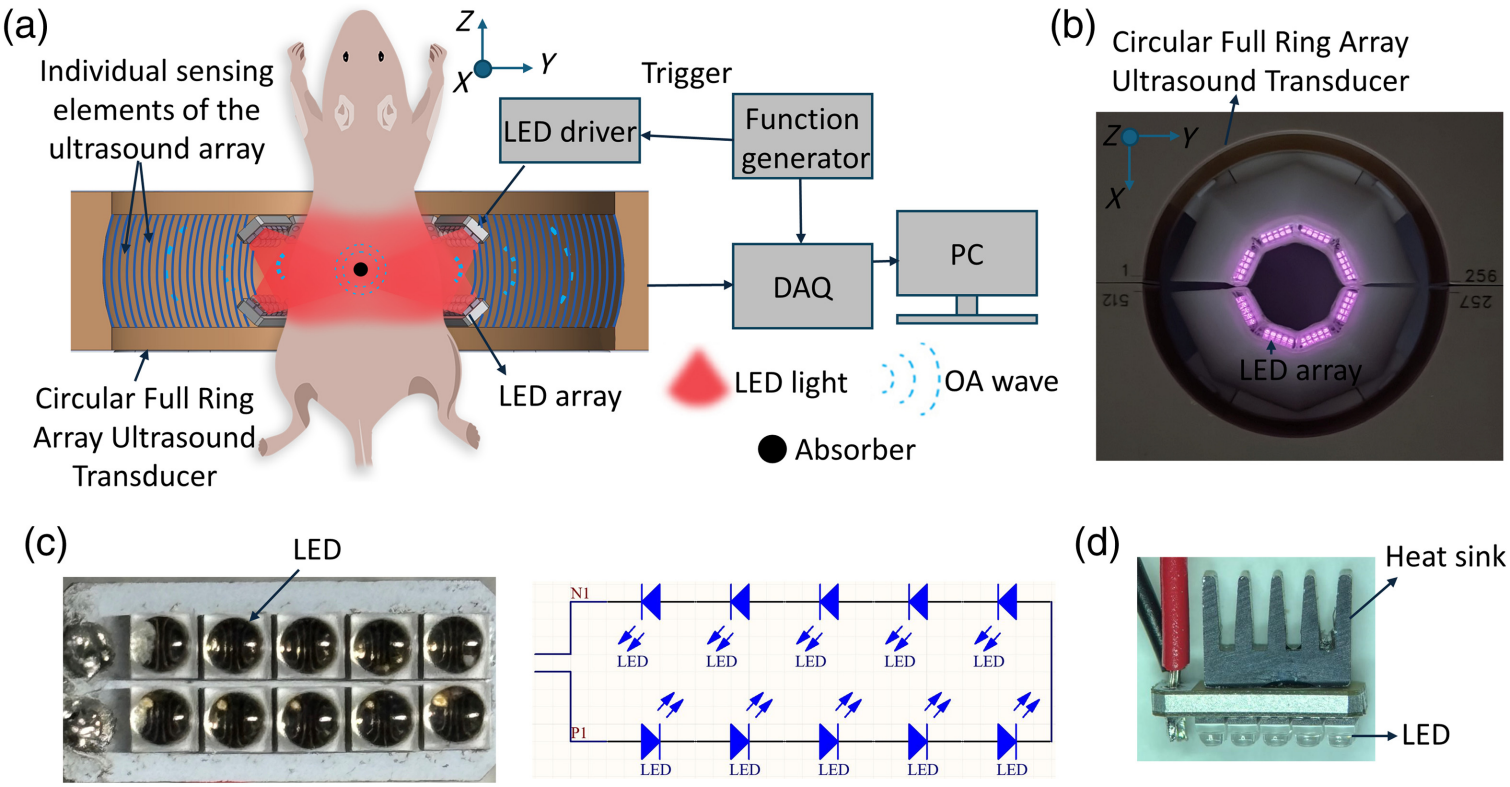
(a) Schematic of full-view light-emitting diode (LED)-based optoacoustic tomography (FLOAT) system for *in vivo* imaging of mice. Individual sensing elements of the ring array are shown with cylindrical lines on the inside of the surface of the array transducer. DAQ, data acquisition unit; OA, optoacoustic; PC, personal computer. (b) Photograph of circular full-ring array transducer together with LED arrays augmented to the bottom of the array transducer. LED arrays on the top side of the array transducer are not shown. (c) Left: photograph of a single LED array with 10 LEDs connected in series, right: circuit diagram. (d) Photograph of the single LED array with a heat sink attached on the back.

Schematic of the FLOAT system is shown in Fig. 1. It utilizes high-power stacked arrays, each consisting of 10 LEDs (SFH4171S, OSRAM, Munich, Germany), for light excitation at 850 nm wavelength. Sixteen such arrays were incorporated, with eight attached to both the top and bottom of a custom-built circular 80-mm-diameter ultrasound transducer (IMASONIC SAS, Voray-sur-l’Ognon, France). These LED arrays provided uniform illumination around the imaged object. A plexiglass membrane (2 mm thickness) isolated the LED circuit from water while allowing light transmission. The LEDs were pulsed using a custom-designed driver unit^32^ that delivered peak currents of ∼40  A, controlled by an external function generator (Agilent, Santa Clara, California, United States) at 50 Hz and powered by a high-voltage supply (Wemaxpower, Shenzhen, China). Note that the heat generated by the LED arrays in pulsed mode operation is dissipated using heat sinks attached to the back of each LED array [Fig. 1(d)]. The full width at half maximum (FWHM) of the light pulse from LEDs is ∼100  ns with rise and fall times of ∼40 and ∼30  ns, respectively.^32^ Each LED emitted ∼3  μJ per pulse, as measured with a pyroelectric sensor (J-10MT-10 KHZ, Coherent, Saxonburg, Pennsylvania, United States), resulting in a total of 0.48 mJ with a total of 160 LEDs (16 arrays) that corresponds to a peak energy density of $∼51\;\mu J/{\mathrm{cm}}^{2}$ on the skin surface of the mouse.

The circular full-ring ultrasound transducer had 512 elements, each measuring $0.37\times 15\;mm^{2}$ area, with an inter-element pitch of 0.47 mm, having a central frequency of 5 MHz and a nominal bandwidth of over 60%. The elements were distributed on two arcs, each covering a 174-deg angle, and focused at a distance of 38 mm in the elevational direction. Optoacoustic signals were digitized at 40 Mega samples per second using a custom DAQ (Falkenstein Mikrosysteme, Taufkirchen, Germany) and transmitted via 1  Gb/s Ethernet to a PC for storage and processing, controlled through a MATLAB (R2022a) interface.

**Fig. 2:**
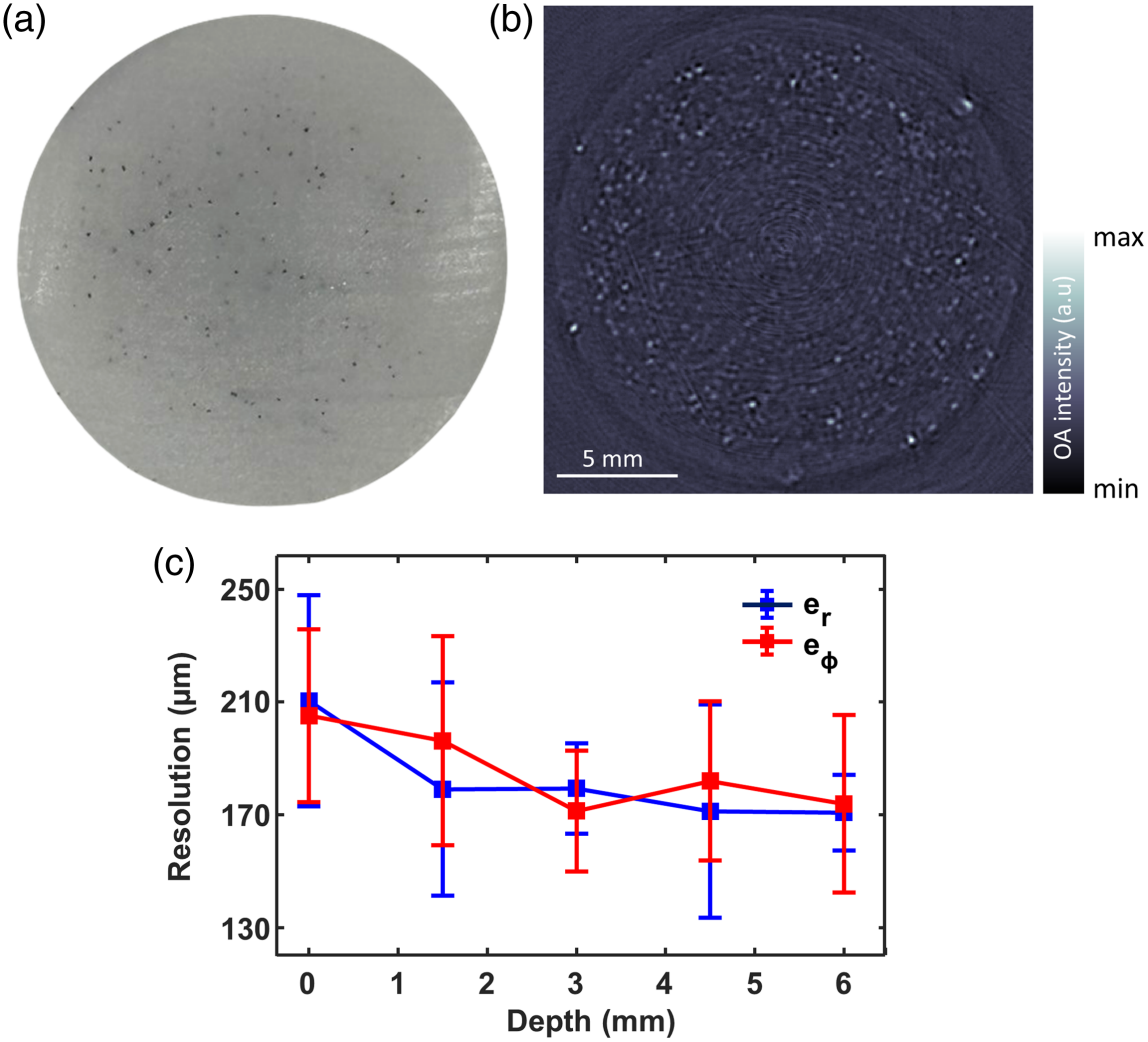
FLOAT system resolution characterization. (a) Photograph of the tissue-mimicking agar phantom (consisting of a mixture of Intralipid and Indian ink to mimic tissue background scattering and absorption) embedding randomly distributed absorbing cloud of 100-μm spheres. (b) The reconstructed cross-sectional 2D OAT image from the sphere phantom. (c) System resolution characterization along the depth dimension. $e_{r}$, radial resolution. $e_{\phi }$, azimuthal resolution.

The spatial resolution of the FLOAT system was characterized by scanning a cloud of 100-μm spheres distributed randomly in a tissue-mimicking phantom [Fig. 2(a)]. Specifically, we used polyethylene microspheres (100-μm diameter; Cospheric Inc., Santa Barbara, California, United States) embedded in a tissue-mimicking agar phantom (20-mm diameter). The phantom consisted of 1.3% agar powder by weight in water, 1.2% by volume of Intralipid®, and 0.002% by volume of black India ink, simulating a background absorption coefficient of $\mu_{a}=0.2\;{\mathrm{cm}}^{-1}$ and a reduced scattering coefficient of $\mu_{s}^{\prime }=10\;{\mathrm{cm}}^{-1}$, typical for biological tissues at an excitation wavelength of 850 nm. The phantom was vertically scanned at 1-mm intervals along the z-direction (RCP2-RGD6c-I-56 P-4-150-P1-S-B, IAI Inc., Shizuoka Prefecture, Japan) to capture different sphere distributions.

All *in vivo* animal experiments were conducted in compliance with the Swiss Federal Act on Animal Protection and were approved by the Cantonal Veterinary Office of Zurich. Female athymic nude-Foxn1^nu^ mice were anesthetized with isoflurane (5% volume ratio for induction and 1.5% volume ratio during the experiments; Provet AG, Lyssach, Switzerland) in a mixture of oxygen and air (100/400  mL/min). The animals were secured in a stable position using a custom-made holder, with both fore and hind paws immobilized. A transparent thin membrane was employed to cover the nose and mouth of the mice, allowing them to breathe gas anesthesia freely, with the head kept above the water surface, while the body was fully submerged in water. The water temperature was maintained at 36°C throughout the experiments using a feedback-controlled heating stick. The mice were scanned in a stop-and-go manner, with 6-mm steps in the vertical direction, using the full-ring ultrasound transducer. The position of the transducer was controlled by motorized stages (RCP2-RGD6c-I-56P-4-150-P1-S-B, IAI Inc., Shizuoka Prefecture, Japan), which allowed translation in the vertical (z) direction.

Several signal-processing steps were applied before the image reconstruction process. First, the collected signals from the imaged object were averaged over 500 consecutive pulses. Subsequently, the averaged baseline signals, obtained when only water was present in the field of view, were subtracted from the averaged signals of the imaged object. A notch filter was then utilized to reduce the abnormal peak frequencies observed in the Fourier transform of the signal matrix. The filtered signal matrix was then used for the OAT image reconstruction, employing a model-based reconstruction algorithm with statistical weighting to minimize artifacts related to acoustic reflections and scattering.^33^

## 3 Results

The reconstructed cross-sectional image of the microspheres phantom for a section containing a large number of spheres is shown in Fig. 2(b). The spatial resolutions along the radial/axial direction ($e_{r}$) and azimuthal/tangential direction ($e_{\phi }$) were estimated at each radial position after deconvolving the actual microsphere diameter from the corresponding full width at half maximum (FWHM) in the image, which was calculated after fitting to Gaussian curves in both directions. The radial and azimuthal resolutions were computed from the mean resolution values of the five brightest spheres at each depth (∼0, 1.5, 3, 4.5, and 6 mm) from the phantom surface [Fig. 2(c)]. The in-plane mean radial resolution and mean azimuthal resolution were in the range of 170 to 210  μm. The resolution improves toward the center of the phantom attributing to the cylindrically focused detection of the ring array transducer. Note that the spheres are clearly discernible up to a depth of 6 mm, which defines the imaging depth across the entire FOV in 360 deg.

**Fig. 3:**
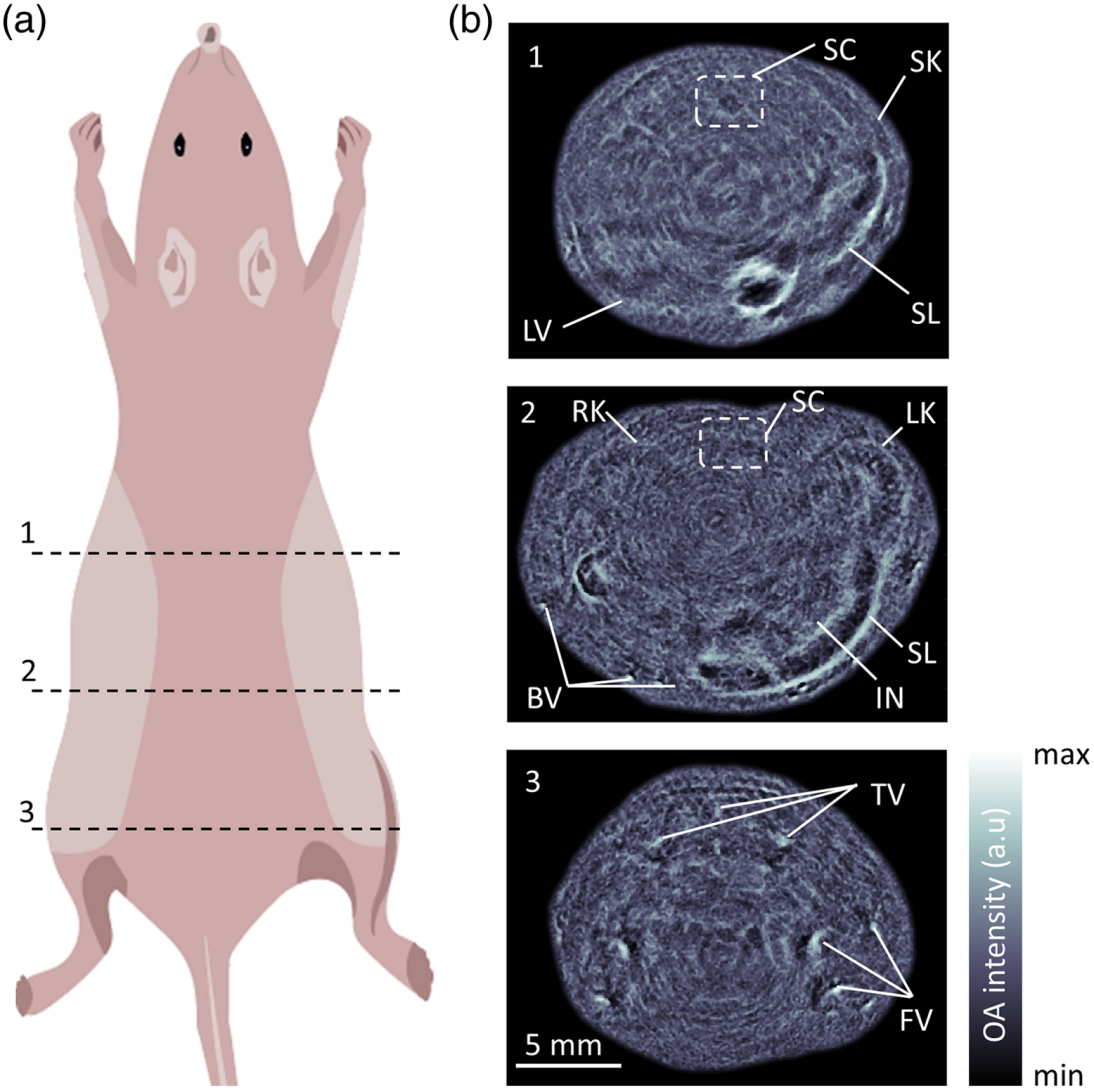
Whole-body *in vivo* imaging of mice with FLOAT: (a) Different cross-sectional planes at which the mouse was scanned. (b) The reconstructed 2D images. SC, spinal cord; SK, skin; SL, spleen; LVL, liver; RK, right kidney; LK, left kidney; IN, intestine; BV, blood vessel; TV, tail vein; FV, femoral vein.

The system’s ability to perform whole-body imaging of mice was subsequently demonstrated (Fig. 3). The immobilized animal was scanned from the abdominal to the tail region [Fig. 3(a)]. The LED arrays together with the circular ring array transducer were translated in steps of 6 mm in z direction along the length of the mouse torso. Three representative cross-sectional (2D) images reveal different anatomical features inside the mouse. Major organs such as the spleen, liver, left kidney, right kidney, spinal cord, and intestines, as well as some fine anatomical structures such as the surrounding vasculature (tail veins, femoral veins) and the skin outline, were clearly discernible [Fig. 3(b)].

## 4 Discussion and Conclusion

Overall, our system provides a safe and compact alternative with a cost of around $1 to 2 k, significantly cheaper than both the traditional Nd: YAG-based laser sources, whose cost often exceeds $100 k, as well as the laser-diode-based solutions. Note that this cost includes only the LED arrays together with the pulse driver modules and does not include the ring array, DAQ, and other receiving electronics. However, LEDs have lower peak power, making them less efficient for optoacoustic signal generation. Yet, the FLOAT system compensates for this by placing LED arrays very close to the imaged object, thus generating sufficient energy density for deep tissue imaging. Previous LED-based systems using linear arrays suffered from severe limited-view artifacts resulting in impaired image quality.^34^ Instead, FLOAT circumvents the limited-view problem with a custom full-ring ultrasound array and 360-deg light illumination, thus facilitating optimal acquisition of tomographic image data.

Mice were imaged away from the heart below the thorax region to minimize motion artifacts. FLOAT was able to render high-resolution cross-sectional images down to 170  μm up to a depth of ∼5  mm. Note that, we have averaged 500 frames at a pulse repetition rate of 50 Hz, acquiring each cross-sectional data within a 10-s duration, which corresponds to an acquisition frame rate of 0.1 Hz for 2D *in vivo* imaging. A sequence of dedicated signal and image processing steps was followed to mitigate the noise- and motion-related artifacts in the images. The image quality can further be improved by increasing the repetition rate to allow for faster scan times, which in turn reduces motion artifacts and minimizes blurring in the reconstructed images.

Future advancements are expected to include multi-spectral imaging capabilities using LEDs of different wavelengths to assess tissue functional parameters, such as blood oxygen saturation, contrast agent perfusion, and targeting. Other applications beyond small animal imaging can also be envisioned, such as the detection of synovial angiogenesis in the finger joints, a key marker of early rheumatoid arthritis.^35^ In addition, the circular-ring array transducer can support both transmission and reflection ultrasound imaging, offering complementary data on the elastic and functional properties of finger joints.^36^^,^^37^ The current FLOAT system’s limited SNR may be improved through advanced reconstruction and processing techniques. For instance, deep-learning methods^38^ could help reduce ring artifacts caused by electrical noise, whereas model-based least squares (LS) minimization techniques could further enhance *in vivo* image quality and mitigate artifacts.^39^

In summary, FLOAT opens up new venues for preclinical OAT studies in resource-limited settings, such as monitoring pharmacokinetics and biodistribution of molecular agents and drugs on a whole-body level.

## Data Availability

The raw dataset used for this study is available upon request to the corresponding author.
